# The complete mitochondrial genome and phylogenetic analysis of *Euurobracon yokahamae* (Hymenoptera: Braconidae)

**DOI:** 10.1080/23802359.2022.2080605

**Published:** 2022-06-07

**Authors:** Wanggyu Kim, Hye-Woo Byun, Mi-Jeong Jeon

**Affiliations:** Animal Resources Division, National Institute of Biological Resources, Incheon, Korea

**Keywords:** *Euurobracon yokahamae*, mitochondrial genome, Braconidae, parasitoid wasp, phylogenetic analysis

## Abstract

*Euurobracon yokahamae* is a parasitoid wasp found solely in Asia, and is endangered in some countries. The complete mitochondrial DNA sequence of *E. yokahamae* was sequenced using next-generation sequencing (NGS). The mitogenome of this species is 14,974bp long and encodes for 13 protein-coding genes (PCGs), 22 transfer RNAs, and 2 ribosomal RNAs. Maximum likelihood phylogenetic analysis of the mitochondrial genome of braconid species was performed. Tree topology showed that *E. yokahamae* was closely related to another species of the same genus.

*Euurobracon yokahamae* (Dalla Torre, 1898) (Hymenoptera: Braconidae) is an ectoparasitoid of Cerambycidae (Coleoptera) and Buprestidae (Coleoptera) larvae that live in wood. It is characterized by an extremely long ovipositor (4–8 the length of its body) (Kaga et al. 2018). It is not registered on the International Union for Conservation of Nature (IUCN) Red List as an endangered or threatened species. Although *E. yokahamae* is protected as a near threatened (NT) species (Ministry of the Environment 2021) in Japan, it has not been established if *E. yokahamae* is a threatened species in South Korea. Further research is required to determine if *E. yokahamae* should be considered an endangered species in South Korea. This study was conducted as part of a research project by the Ministry of Environment of South Korea. Permission to collect a sample in Korea was not required.

A sample of *E. yokahamae* (IV0004) was collected from Paju-si, Gyeonggi-do, South Korea (37°46'36.2"N, 126°51'26.1"E) on 17 July 2020. The specimen was deposited at the National Institute of Biological Resources (NIBR; Incheon, South Korea) (https://www.nibr.go.kr/cmn/main/enMain.do, Mi-Jeong Jeon: jeonmj@korea.kr) under the voucher number TZVNGR0000000002. Genomic DNA (gDNA) was extracted using a blood and tissue DNA extraction kit (QIAGEN, Germantown, MD) and amplified using the REPLI-g kit (QIAGEN) to the appropriate concentration for sequencing. The fragment required for Illumina DNA PCR-Free library preparation was approximately 350 bp. Next-generation sequencing was performed on the IlluminaNovaSeq6000 platform at Theragen Bio Inc. (Gyeonggi-do, South Korea). The mitogenome sequence arrangement and annotation were performed using Geneious Prime 2021.1.1 (Biomatters, Auckland, New Zealand).

The complete mitochondrial genome sequences of *E. yokahamae* (OL825724) was 14,974 bp Long, and contained 13 protein-coding genes (PCGs), 22 transfer RNA genes (tRNA), and 2 ribosomal RNA (rRNA) genes. The overall nucleotide compositions were 45.2% A, 43.5% T, 5.2% C, and 6.1% G. All PCGs had the stop codons ‘TAA’. Six of the PCGs (*COX2, ATP8, ATP6, ND3, ND5, ND1*) had the start codon ‘ATT’. Five of the PCGs (*COX1, COX3, ND4, ND6, CYTB*) had the start codon ATG. The remaining PCGs, ND2 and ND4L had the start codon ‘ATA’.

The PhyML 3.0 program was used to construct a maximum likelihood tree using the general time reversible model (Nei and Kumar 2000) which incorporated invariant sites with gamma distribution (GTR + I + G) (Guindon and Gascuel 2003; Guindon et al. 2010). The most suitable maximum likelihood model was chosen in jModeltest 2.1.9 (Posada 2008) using the Akaike information criterion. Phylogenetic analysis was performed using the 13 PCGs and the 2 rRNAs of the mitochondrial genome of 12 Braconidae species, including one *Euurobracon* species. Sequences were aligned using MAFFT version 7 using the G-INS-I method (Golubchik et al. 2007). *Trichopria drosophilae* (Perkins, 1910) (Diapriidae) was used as an outgroup to root the tree. The phylogenetic analysis showed that *E. yokahamae* was closely related to *Euurobracon breviterebrae* Watanabe, 1934 (Figure 1).

**Figure 1.**
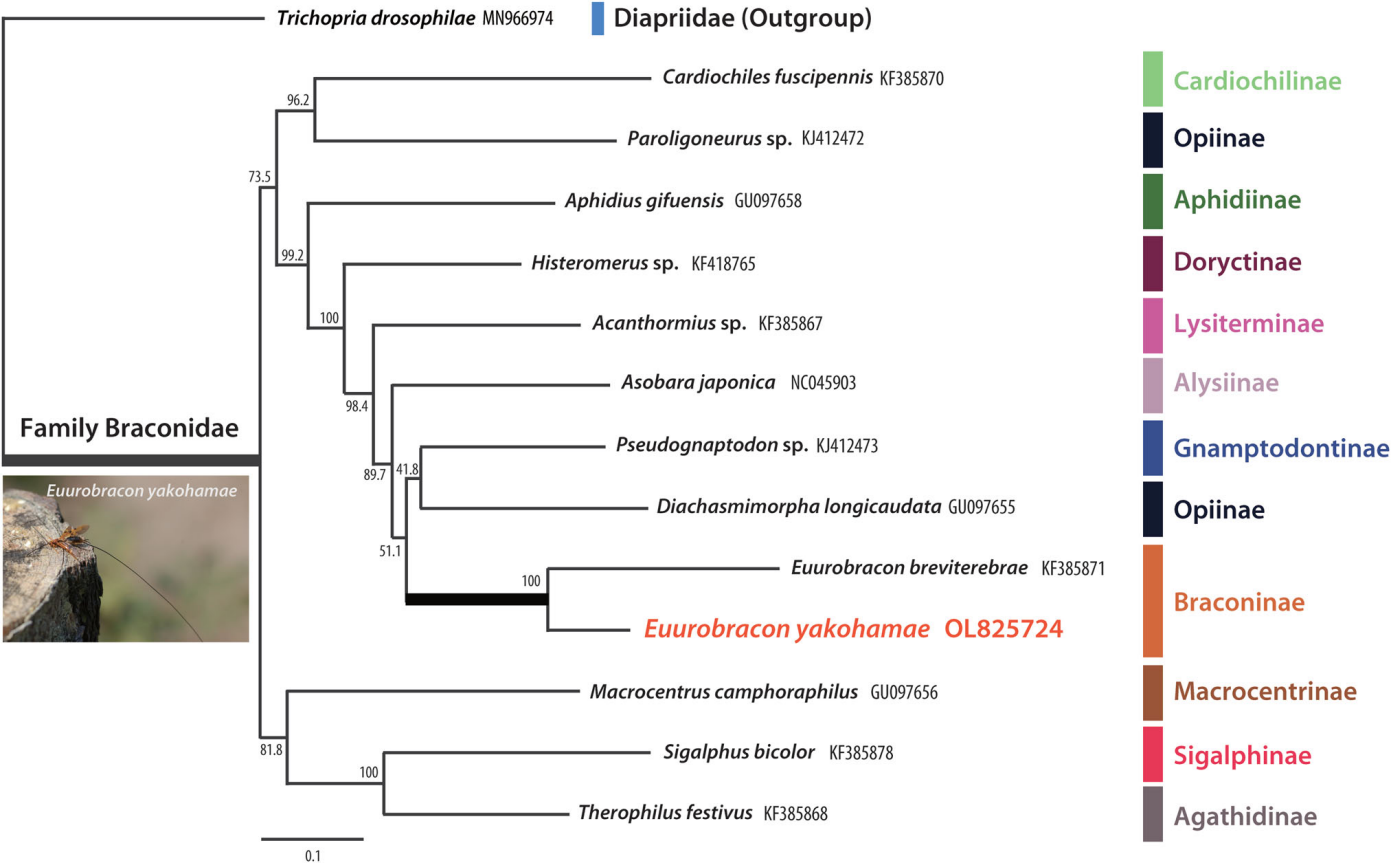
Maximum-likelihood (ML) phylogenetic tree based on 13 PCGs and 2 rRNAs of 13 Braconidae mitogenomes. Numbers at nodes indicate bootstrap values for each node. Trichopria drosophilae was used as an outgroup.

## Data Availability

The genome sequence data that support the findings of this study are vailable in the NCBI GenBank database (https://www.ncbi.nlm.nih.gov) under accession no. OL825724. The associated BioProject, SRA, and Bio-Sample numbers are PRJNA803924, SUB11040386 and SAMN25690371, respectively.
